# Supplementary material for: Muscarinic receptors mediate motivation via preparatory neural activity in humans
Source: eLife. 2024 Nov 20;13:RP98922. doi: 10.7554/eLife.98922 (PMC11578592; doi:10.7554/eLife.98922)
Supplement: Figure 3—source data 2. — Pre-preparation cue activity and P3a did not predict any behavioural measure, while CNV predicted reaction times (RT) and distractor pull (Bonferroni-corrected threshold: α=0.0056). The regression formula controlled for all factors and their interactions, and a random effect of participant: ‘ERP ~ 1 + behaviour + incentive * distractor * THP + (1 | participant)’. Significant effects are shown in bold italics. [file elife-98922-fig3-data2.zip › Figure 3 Source Data 2.docx]

| ERP | Behaviour | β | CI | SE | t | p |
| --- | --- | --- | --- | --- | --- | --- |
| P3a | Residual velocity | 0.0022 | -0.0125, 0.0170 | 0.0075 | 0.2967 | .7667 |
|  | RT | 0.0120 | 0.0004, 0.0236 | 0.0059 | 2.0313 | .0422 |
|  | Distractor pull | 0.0074 | -0.0068, 0.0215 | 0.0072 | 1.0225 | .3066 |
| CNV | Residual velocity | 0.0150 | 0.0002, 0.0298 | 0.0076 | 1.9811 | .0476 |
|  | ***RT*** | ***0.1282*** | ***0.1165, 0.1398*** | ***0.0059*** | ***21.5854*** | ***< .0001*** |
|  | ***Distractor pull*** | ***0.0688*** | ***0.0544, 0.0831*** | ***0.0073*** | ***9.4145*** | ***< .0001*** |
| Incentive cue ERP | Residual velocity | -0.0054 | -0.0210, 0.0102 | 0.0080 | -0.6758 | .4992 |
|  | RT | -0.0030 | -0.0156, 0.0096 | 0.0064 | -0.4626 | .6437 |
|  | Distractor pull | 0.0100 | -0.0049, 0.0250 | 0.0077 | 1.3132 | .1891 |
